# A genetic tool for production of GFP-expressing *Rhodopseudomonas palustris* for visualization of bacterial colonization

**DOI:** 10.1186/s13568-019-0866-6

**Published:** 2019-09-10

**Authors:** Zhongying Zhai, Jiao Du, Lijie Chen, Muhammad Rizwan Hamid, Xiaohua Du, Xiaoting Kong, Jue Cheng, Wen Tang, Deyong Zhang, Pin Su, Yong Liu

**Affiliations:** 1 Longping Branch, Graduate School of Hunan University, Changsha, 410125 China; 2 Hunan Plant Protection Institute, Hunan Academy of Agricultural Sciences, Changsha, 410125 China

**Keywords:** *Rhodopseudomonas palustris*, Green fluorescent protein, Colonization, CLSM, Visualization

## Abstract

Development of a genetic tool for visualization of photosynthetic bacteria (PSB) is essential for understanding microbial function during their interaction with plant and microflora. In this study, *Rhodopseudomonas palustris* GJ-22-gfp harboring the vector pBBR1-pckA_PT_-gfp was constructed using an electroporation transformation method and was used for dynamic tracing of bacteria in plants. The results showed that strain GJ-22-gfp was stable and did not affect the biocontrol function, and the Confocal Laser Scanning Microscopy (CLSM) results indicated it could successfully colonised on the surface of leaf and root of tobacco and rice. In tobacco leaves, cells formed aggregates on the mesophyll epidermal cells. While in rice, no aggregate was found. Instead, the fluorescent cells colonise the longitudinal intercellular spaces between epidermal cells. In addition, the results of strain GJ-22 on the growth promotion and disease resistance of tobacco and rice indicated that the different colonization patterns might be related to the bacteria could induce systemic resistance in tobacco.

## Introduction

*Rhodopseudomonas palustris* is a Gram-negative non-sulfur purple bacterium that can propagate through budding and can grow under anaerobic condition in the light. To date, *R. palustris* is widely used in the aquaculture industry, hazardous material degradation, wastewater treatment, livestock and poultry production, and environment protection (Wang et al. 2008; Deyong et al. 2005; Tao et al. 2013; Li et al. 2005). A previous study has also demonstrated that *R. palustris* can be used to improve soil fertility, plant growth, and plant disease resistance (Su et al. 2017). *R. palustris* is considered to be beneficial for sustainable agriculture because it is non-toxic to humans and animals and does not produce toxic residues in soil and water. In addition, *R. palustris* and other photosynthetic bacteria can be used to promote degradation of pesticides and removal of heavy metals from soil during crop production (He 2007).

The activities of bacteria on their host plants and the mechanism of how to promote plant growth and disease resistance are poorly understood, mainly due to lack of powerful and valuable tools to monitor their colonization during the infection cycle in plants. Development of a genetic tool for visualization is essential for understanding the microbial function during their interaction with plants and microflora. Many reports have suggested using specific bacterial antibiotic resistance as markers to track the survival and colonization pattern of *R. palustris* strains in plants and animals (Aarts et al. 2001; Flint et al. 1989). Although the methods using antibiotic markers are simple, rapid and low cost, the antibiotic markers are unstable and easy to lose during the study (Nairn and Chanway 2002; Torres et al. 2013). Also, these methods could not exclude the effects generated by contaminated bacterial strains.

To establish a powerful and valuable tool for the study of *R. palustris*, a green fluorescent protein (*gfp*) gene was introduced into the genome of *R. palustris* to track the bacterial dynamic colonization in plants. Using *gfp* as a marker to investigate protein function in cells or to track pathogen spread in plants or animals has attracted considerable attentions since the gene was cloned from *Aequorea victoria* (Prasher et al. 1992; Niedenthal et al. 1996; Inouye and Tsuji 1994). In 1997, Tombolini et al. (1997) constructed a *gfp* expression cassette tagged *Pseudomonas* bacteria to monitor the colonization, spatial distribution and survival of these microorganisms. Bloemberg et al. (1997) constructed plasmids which expressed a bright mutant of *gfp* in *Pseudomonas* spp. strain WCS365 to demonstrate the association of bacteria with tomato seedling roots. Chen et al. (2005) used *gfp* as a molecular marker to monitor *Bacillus brevis* survival in soil. In 2016, Teh et al. (2016) engineered a gfp-tagged bacterium *E. munditi* to observe its colonization in *S. littoralis.* These studies indicated that *gfp* can be used as a useful and stable marker for visualization of bacterial colonization. However, due to lack suitable vectors, the construction of gfp-tagged *R. palustris* has not been successful. In this study, the gfp-tagged strain GJ-22-gfp along with the gene promoter and terminator of *R. palustris* was constructed using an electroporation transformation method. Expression of *gfp* in this transgenic GJ-22 strain was stable and did not affect the biocontrol function. CLSM results indicated *R. palustris* GJ-22 gives a different colonise pattern on the surface of leaves and roots in tobacco and rice, and the results of later experiments indicated that this might due to the strains induce plants to produce induced resistance. In a word, the construction of gfp-labeled *R. palustris* would greatly facilitate the understanding of the interaction of plant–microbial, which will be of historic significance for the future study.

## Materials and methods

### Bacterial strains, culture conditions and plasmids

The bacterial strains and plasmids used in this experiment are listed in Additional file 1: Table S1. *R. palustris* GJ-22 strain (CGMCC: 17356) were grown in the medium followed Song (2007) with minor modification, named PSB medium. The medium comprised: 0.5 g [NH4]_2_SO_4_, 0.5 g NaAc, 1 g K_2_HPO_4_, 0.05 g FeSO_4_, 0.05 g H_3_BO_3_, 0.05 g Na_2_MoO_4_ and 1.5 g yeast extract dissolved in 1-l of distilled water. The growth medium was adjusted to pH 7.3 then autoclaved for 50 min at 121 °C. *R. palustris* GJ-22 were grown in anaerobic condition under light without antibiotics. *Escherichia coli* were cultured in Luria Bertani (LB) medium at 37 °C with shaking at 200 rpm overnight (Deininger 1989). *Magnaporthe oryzae* strain 70-15 was obtained from the Fungal Genetics Stock Centre, Kansas City, KS, USA. The strain was maintained on CM medium (Talbot et al. 1993). Agar was also added to medium to reach 1.5 g L^−1^ when sub-cultured. Kanamycin (50 mg mL^−1^) or ampicillin (100 mg mL^−1^) was also added to the medium when required. Strain GJ-22 was cultured anaerobically in a two-layer medium plate with 1.3% agarose in the lower layer and 1.8% agarose in the top layer.

### Construction of strain GJ-22-gfp

**Fig. 1:**
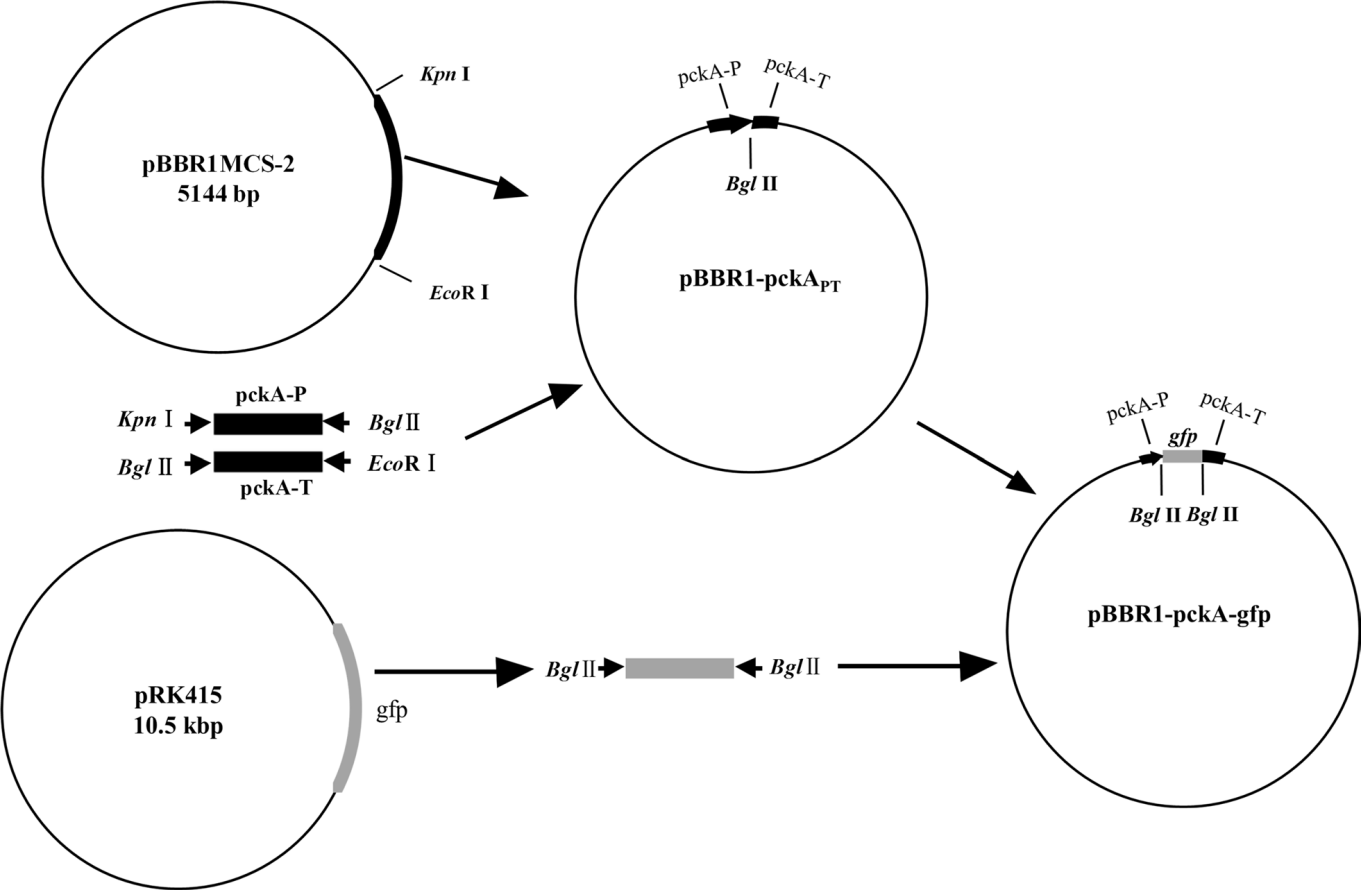
Construction of gfp-tagged plasmid. Plasmid pBBR1MCS-2 was used as cloning vector to express *GFP* under the control of *pckA* promoter of *Rhodopseudomonas palustris.* Restriction sites were shown in “Materials and methods”. The pckA-P and pckA-T were amplificated from the *R. palustris* GJ-22, they were constructed with *GFP* protein as the promoter and terminator to express green fluorescent protein in *R. palustris* GJ-22

*Rhodopseudomonas palustris* GJ-22 strain was inoculated at a rate of 1% inside a flask and kept at 30 °C for 3 days under 7500 lx light. After genomic DNA of *R. palustris* GJ-22 was extracted using the CTAB method with OD_660_ at 1.0 (Doyle 1991), the resulting genomic DNA was checked in 1% agarose gels through electrophoreses. Promoter (pckA-P) and terminator (pckA-T) of *Phosphoenolpyruvate carboxykinase* (*pckA*) gene (Inui et al. 1999) were PCR amplified from the DNA of *R. palustris* GJ-22 using primers pckA-P F/R and pckA-T F/R (primers were show on Additional file 1: Table S2, and the sequences of *pckA* were show on Additional file 2). The primer of pckA-P F and pckA-T R were digested with *Kpn* I and *Bgl* II restriction enzymes respectively, and the primer pckA-T F and pckA-T R were digested with *Bgl* II and *Eco*R I restriction enzymes respectively. These two digested PCR products were first ligated together at the *Bgl* II site and then inserted into the pBBR1MCS-2 vector (GenBank sequence: U23751) at the pre-digested *Kpn* I and *Eco*R I sites to generate pBBR1-pckA_PT_ (Fig. 1). This vector was propagated in the Trans1-T1 competent cells (TransGen Biotech technology, Beijing, China) and plasmid isolation using a plasmid extraction kit (TransGenBiotech, BeiJing, China).

A green fluorescent protein (*gfp*) gene (GeneBank: KP294375.1) was PCR amplified from vector pRK415 with primer gfpmut3 F/R (primers were show on Additional file 1: Table S2, and the sequences of *gfp* were show on Additional file 2). The PCR products were purified (OMEGA Bio-tek, USA), then cloned into the pEasy-T1 vector, resulting vector (pEasy-T1-gfp) was transformed into the Trans1-T1 cells for propagation. The *gfp* gene fragment was released from the pEasy-T1-gfp vector through overnight *Bgl* II enzyme digestion at 37 °C, isolated using a gel extraction kit (OMEGA Bio-tek, USA), and then inserted between the *pckA* promoter (pckA-P) and *pckA* terminator (pckA-T) of vector pBBR1-pckA_PT_ to generate pBBR1-pckA_PT_-gfp (sequence of pBBR1-pckA_PT_-gfp was shown in Additional file 3). The integrity of the pBBR1-pckA_PT_-gfp vector was sequenced by the TsingKe Biological Technology (ChangSha, China). This vector was further propagated in *E. coli* BL21 (DE3) cells (TransGen Biotech technology, Beijing, China) for *gfp* expression.

*GFP* expression in *E. coli* BL21 (DE3) cells were induced by addition of isopropyi-d-thiogalactopyranoside (IPTG) into the culture medium as previously described (Lewis and Marston 1999). The *gfp* expression of IPTG-treated cells was examined under a confocal fluorescent microscope with 488 nm excitation and collecting fluorescence in the range 500–550 nm (Nikon C2 plus, Nikon, Japan). The cells were photographed with Nikon CFI Plan Apochromat VC 60XH, NA:1.4, WD: 0.13 mm. Competent *R. palustris* GJ-22 cells were prepared as previously described (Pelletier et al. 2008). Briefly, *R. palustris* GJ-22 culture was grown under anaerobic condition till the OD_660_ at 0.4, and then cells were pelleted by centrifugation at 13,751*g* for 10 min at 4 °C. The pellet cells were rinsed three times with ice-cold sterile water and then resuscitated in a 10% glycerol solution. Finally, cells were stored in 1.5 mL sterile centrifuge tubes at − 80 °C immediately. Before transformation, the frozen *R. palustris* cells (50 µL) were thawed on ice for 20 min and mixed with 5 ng of pBBR1-pckA_PT_-gfp DNA and 80 μL of sterile ice-cold distilled water inside a 2 mm Gene Pulser cuvette. After 10 min incubation on ice, electroporation was performed using the Eppendorf Eporator (Eppendorf North America, Hauppauge, NY, USA) set at 2.5 kV voltage, 25 µF capacitance, and 100 Ω resistance. After electroporation, the cells were incubated 2 min at room temperature, then grown in 10 mL culture medium for 20 h at 30 °C under light. Positive transformants were selected on agar medium containing kanamycin (50 mg mL^−1^) and cultured for 4 days at 30 °C under light.

### Quantification of *gfp* expression and stability of strain GJ-22-gfp

GJ-22-gfp cells were determined using a confocal fluorescent microscope with 488 nm excitation and collecting fluorescence in the range 500–550 nm (Nikon C2 plus, Nikon, Japan). Images of the cells were captured using a Nikon CFI Plan Apochromat VC 60XH (Nikon, Japan) and processed using the Photoshop 7.0 software for stitching.

Stability of strain GJ-22-gfp was tested by growing the cultures through 15 consecutive passages on the solid culture medium with and without kanamycin. Briefly, for each passage, the GJ-22-gfp cells were grown at 30 °C for 4 days in the liquid medium without antibiotics, and then the cells were inoculated to agar medium plates with and without kanamycin. Colonies on each plate were calculated and then examined under a fluorescence microscope (Olympus BX51, Japan). This experiment was repeated three times.

The growth curves of wild type and gfp-labeled strain were measured to determine whether the addition of *gfp* affects the growth metabolism. Wild type strain GJ-22 and GJ-22-gfp were cultured in the same condition. The cells were centrifuged at 8000*g* and resuscitated with sterile liquid medium when the cells were grown at OD_660_ 1.0. The concentration of cells was adjusted to OD_660_ at 1.0. Diluted cells were inoculated to fresh liquid medium and grown at 30 °C with 7500 lx. The cell cultures were measured for cell concentrations at 12 h post culturing and then once every 12 h till 108 h to generate the cell growth curves. This experiment was repeated three times.

To determine the plasmid stability in GJ-22-gfp during the plant surface colonization and effect of plasmid on strain’s ability for plant surface colonization, bacterial cells were sampled with the following method at the indicated post-inoculation times. Five-week-old tobacco and rice were inoculated using the method of foliar spray and root irrigation with 10 mL wild type strain GJ-22 and GJ-22-gfp (5 × 10^7^ CFU mL^−1^). The third and fourth leaves (down from the top) and roots (5 cm) of tobacco and rice were harvested from individual assayed plants and pooled at 0th, 24th, 48th, 72th h. Five gram tissues were sampled from each harvested sample and submerged in 100 mL PBS conical flask for 20 min. Samples were then sonicated for 10 min at 47 kHz with 150 rpm shaking. Bacterial cells were pelleted from each sample by 10 min centrifugation at 10,000*g*. The pelleted cells were resuscitated with 2 mL PBS buffer and remove the remaining plant debris by 12,000*g* centrifugation for 10 min.

The bacterial population density of GJ-22 and GJ-22-gfp in phyllosphere and rhizosphere of tobacco and rice were determined by plating the serially cell samplings on the PSB agar medium. The serially diluted GJ-22 cells were platted on the PSB agar medium without kanamycin, and the serially diluted cells of strain GJ-22-gfp were platted on the PSB agar medium with and without kanamycin respectively. Plates were subjected to culture for 5 days before the colony-forming units (CFU) on each plate were calculated.

### Plant growth conditions and colonisation visualization

Seeds of tobacco cv. (*Nicotiana benthamiana* cv. Changsha, collected from the experimental research station) and rice seeds (Longping seeds, Changsha) were obtained from horticultural crop pest control laboratory of Hunan, and these seeds were sterilized with 75% ethanol for 5 min, then rinsed with sterile water thoroughly. Four treatments were set in this experiment: GJ-22 suspension, GJ-22-gfp suspension, PSB medium and ddH_2_O were as the control blank (CK). To observe whether the insertion of exogenous gene would influence the interaction effect between bacteria and plant, the root length and dry mass of tobacco and rice were examined. The treatments was immersed in seeds for 24 h, and the seeds were placed in a wet filter paper culture dish and germinated at 25 °C under red light. Three dishes of each treatment and 100 seeds of each dish were cultured with liquid after germination. Root length and dry mass of tobacco and rice were measured on the 14th and 21th day, respectively.

For visualization the colonization dynamics, 5-week-old tobacco and rice were root-irrigated with *R. palustris* GJ-22-gfp (5 × 10^7^ CFU mL^−1^) by pouring 15 mL of *R. palustris* GJ-22-gfp culture into each pot. In a separate experiment, the same volumes of *R. palustris* GJ-22-gfp were sprayed onto leaves of plants. The equal volumes of medium were used as the control. At 24th, 48th and 72nd day post inoculation (dpi), the assayed plants were harvested, rinsed with sterile phosphate buffered saline (PBS) solution, and the fresh leaves and roots were collected to examine the *R. palustris* GJ-22-gfp colonisation under the confocal microscope. Images were captured under the confocal microscope equipped with a 20× objective (N.A–0.75) and a 488 nm emission and collecting fluorescence in the range 500–550 nm.

### Detection of plants growth promotion

Based on the results of CLSM, it is hypothesized that the GJ-22 could formed bacterial aggregates in tobacco leaves, which could possess ISR-inducing properties. *Nicotiana benthamiana* (TMV systematic host) and rice seeding were cultured 7 days under red light, and then transplanted into single pot. After cultured 7 days, the plants with the same growth condition were selected to divide into four groups, and 30 plants were used for each treatment. These treatments were as following: (i) Spraying the leaves with 5 mL of bacterial suspension (10^8^ CFU mL^−1^); (ii) root irrigation with 5 mL bacterial suspension (10^8^ CFU mL^−1^); (iii) treat plants with the same volume of medium (foliar spray and root irrigation); (iv) treatment plants with the same volume of ddH_2_O as the blank control. The leaf treatment was foliar spray to shoot of tobacco and rice to soaring wet. Dry mass of tobacco and rice were quantified at 1st, 2nd, 3rd and 7th day after disposed. The dry mass of each plant were weighted after drying at 60 °C for 3 days. This experiment was repeated three times.

### TMV and *Magnaporthe oryzae* determination

TMV accumulation was tested by enzyme-linked immunosorbent assay (ELISA), the treatment was according the manufacturer instruction (IBL-America, Minneapolis, USA). And the blast lesions of *M. oryzae* were counted in rice leaves. Tobacco and rice were pre-treated with the same treatment as plants growth promotion: foliar spray to shoot, root irrigation, medium and ddH_2_O respectively after cultured 4 weeks. TMV particles and *M. oryzae* were inculcated the next day of treatment. The samplings were collected at the time of 1st, 2nd, 3rd and 7th day after the tobacco inoculated with 20 µL TMV particles (10^−5^ mg mL^−1^) and the rice inoculated with 5 mL of *M. oryzae* (4.0 × 10^5^ CFU mL^−1^). For tobacco, 1 g fresh leaf tissue of inoculated top blade from each different treatments and different times were collected, flash-frozen and grind with 1 mL of PBS buffer (pH = 7.4), then centrifuged with 8801*g* for 20 min. The suspensions were detected by ELISA, and the TMV accumulation was determined with a microplate spectrophotometer at 450 nm. For rice, samplings were obtained to count the blast lesions of *M. oryzae* of in per unit area.

### Statistical analysis

All the experiments performed in this study were repeated three times. Bacterial population data were log transformed before subjected for further analysis. Statistical differences between treatments were determined by the Tukey’s test using the software of SPSS statistics 17.0 (IBM Corp., New York, USA).

## Results

### Construction of *gfp*-labeled strain

The inserted *pckA*-*gfp* gene was confirmed through sequencing and gel detection, result showed a bright and single band at 750 bp. Expression of *gfp* in *E. coli* showed the strong green fluorescence. The positive GJ-22-gfp colonies were examined under the confocal fluorescence microscope. Results showed that almost all of transformed *R. palustris* GJ-22 cells showed a strong green fluorescence signal (Additional file 1).

**Fig. 2:**
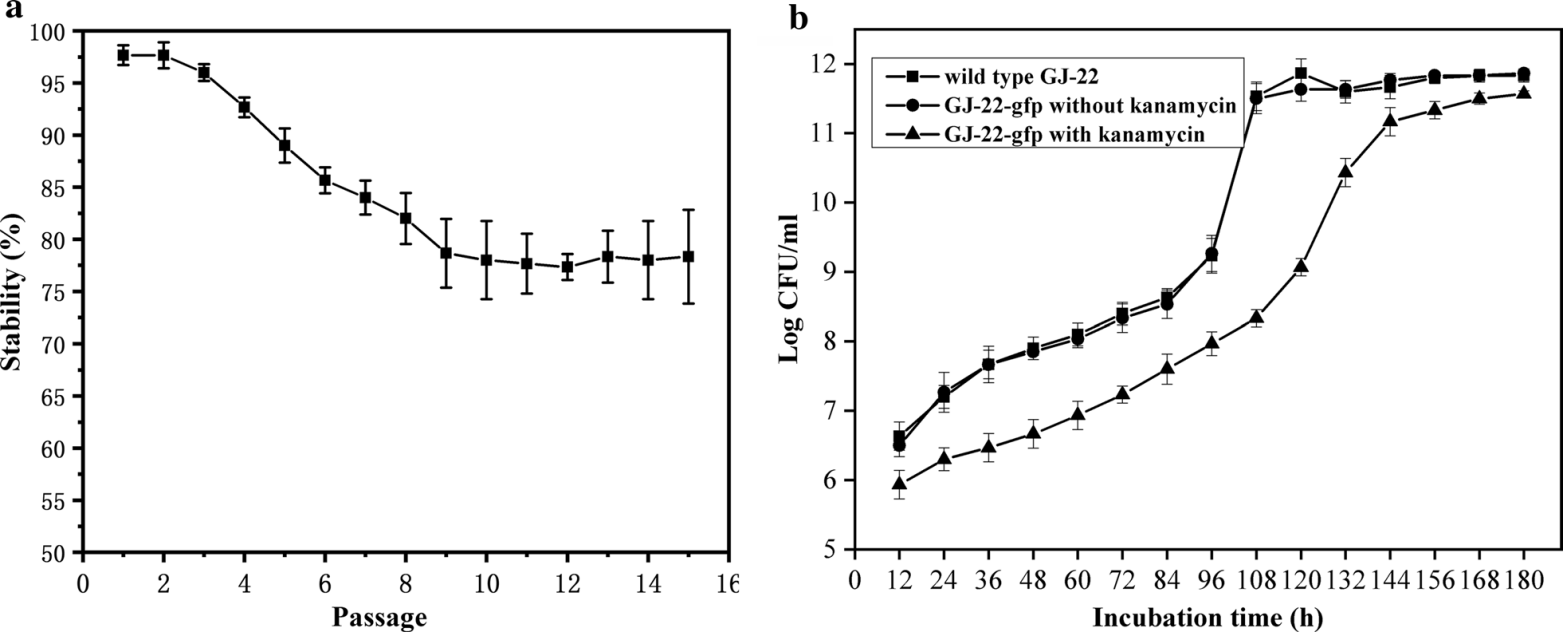
**a** Genetic stability of strain GJ-22-gfp. Strain GJ-22-gfp was applied to PSB medium with and without kanamycin, samples were required every 96 h, repeated 15 times, and each time repeated 3 times. Comparative observation the number of transformants growing on selective and nonselective plates, and at the same time, both plates were observed under fluorescence microscope. After five passages, the maintenance was 90%. After nine passages, the stability of labeled strain maintained to 79%. **b** The growth curves of strain GJ-22 and GJ-22-gfp. Population expressed in log CFU mL^−1^, the figure square represents the wild type strain GJ-22, results indicates the strain GJ-22-gfp without kanamycin, and the triangle was the symbol of the strain GJ-22-gfp with kanamycin. Three strains were cultured in the same media at the same conditions. This experiment repeated three times

To detection the stability of gfp-labed strain, strain GJ-22-gfp were cultured and examined for *gfp* expression through 15 passages in the culture medium with and without kanamycin. The result showed that about 10% of the colonies lost their *gfp* signal after five passages, and the stability rate was maintained at 79% after nine passages (Fig. 2a).

**Fig. 3:**
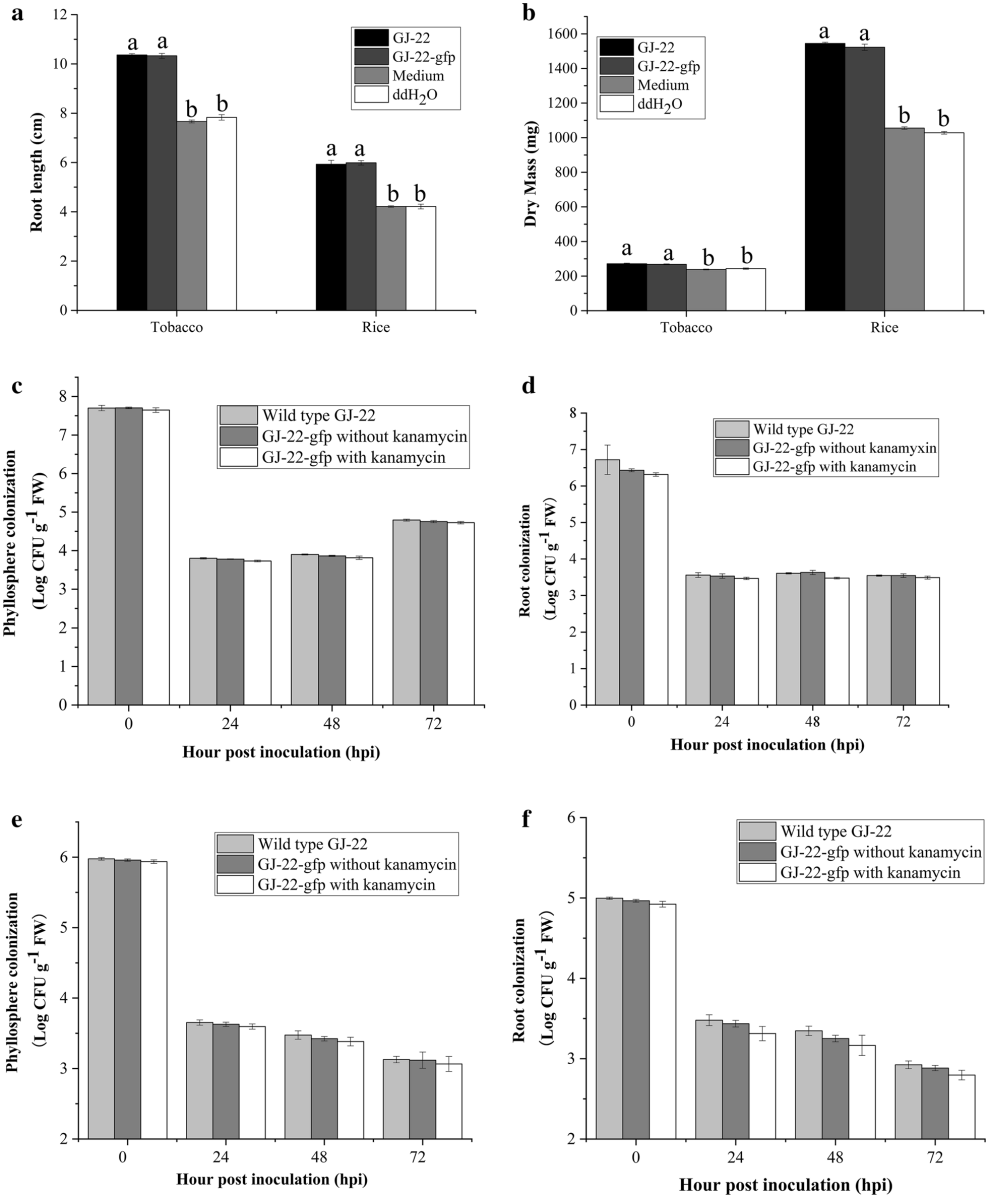
The growth promotion of strain GJ-22 and GJ-22-gfp in tobacco and rice (A,B). Root length (**A**) and dry weight (**B**) of seeding in tobacco and rice inoculated with four treatments. GJ-22, GJ-22-gfp, medium and ddH_2_O. And the different letters indicate significance of treatments (P < 0.05). The population of strain GJ-22 and GJ-22-gfp colonised on the phyllosphere and rhizosphere of tobacco (**C**, **D**) and rice (**E**, **F**). The strain GJ-22 were cultured on the agar medium without kanamycin, and the strain GJ-22-gfp were cultured on the medium with and without kanamycin respectively (P > 0.05)

Growth curves of the wild type GJ-22 and transgenic GJ-22-gfp were determined by growing them in liquid medium without kanamycin. Results showed that the wild type and transgenic *R. palustris* GJ-22 grew similarly in the liquid medium without kanamycin. However, with the addition of kanamycin, the growth of GJ-22-gfp cells was lagged (Fig. 2b). This result indicated that insertion of pBBR1-pckA_PT_-gfp into *R. palustris* GJ-22 did not affect the growth, but with kanamycin, the growth of GJ-22-gfp was lagged. In identifying the biocontrol function of labeled strains, results showed that strain GJ-22-gfp could increase the root length and dry mass of rice and tobacco as wild type strain GJ-22, both higher than the blank control significantly, and no significant differences (P < 0.05) between the strain GJ-22-gfp and the wild type strain GJ-22 (Fig. 3A, B).

To determine the plasmid stability during plant surface colonization, the tagged-strain cells were washed off from plant surfaces at multiple time-points and cultured on plate medium with and without kanamycin. The results showed that there were no significant differences between the population sizes from the two plates, indicating the plasmid functioned stably during the plant surface colonization of the strain GJ-22-gfp (Fig. 3C–F).

The effect of plasmid on the strain´s ability to colonise plant surface as determined by comparing the population size between the wild type strain and tagged strain. During the bacterial colonization on plant surface, there was no significantly different population size detected between the two strain, indicating that the plasmid had no detectable influence on strain’s ability for plant surface colonization (Fig. 3C–F).

### CLSM of visualization the colonization of GJ-22-gfp in tobacco and rice

**Fig. 4:**
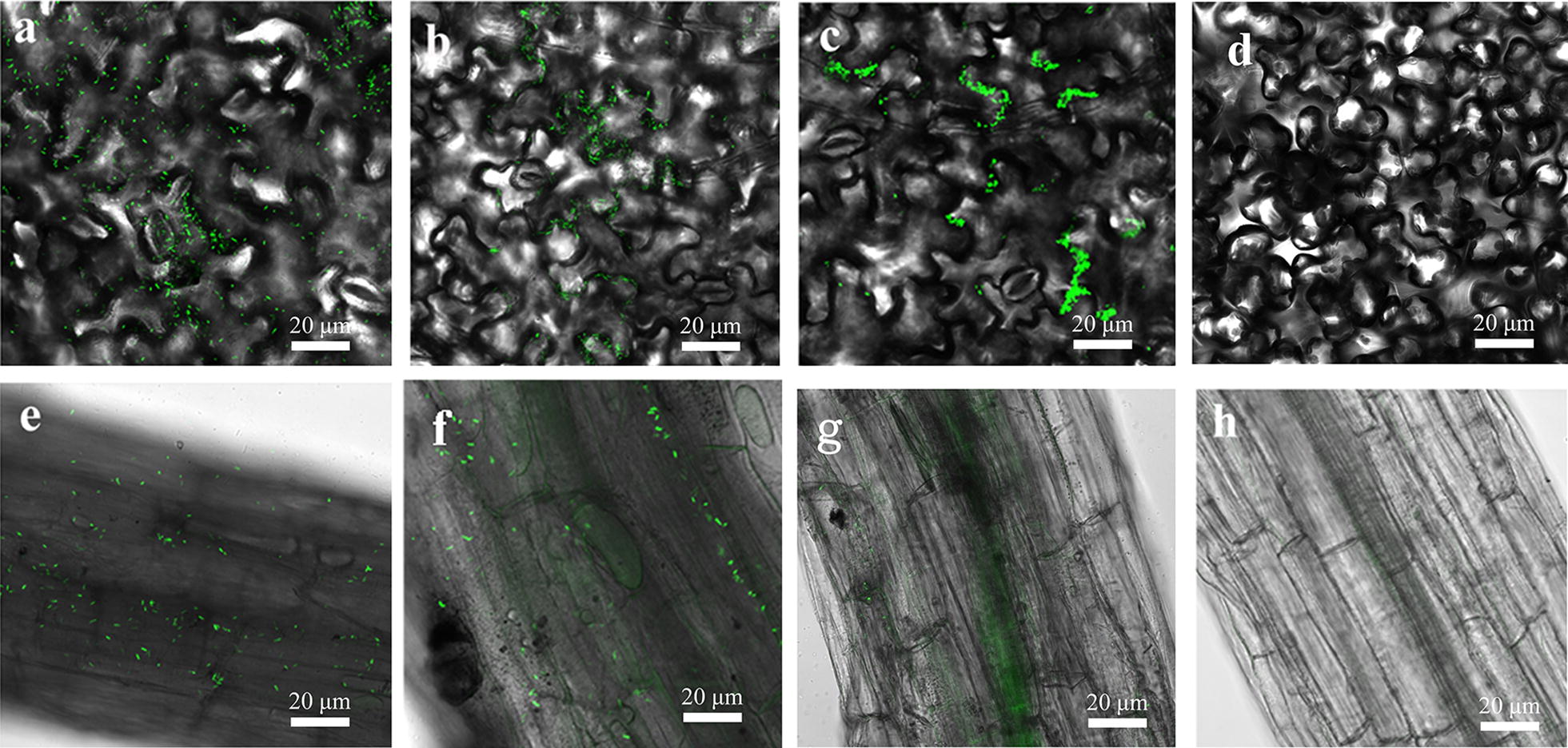
CLSM images of colonization of strain GJ-22-gfp on the leaves and root of tobacco. **a**–**c** and **e**–**g** were the results of foliar and root treatment for 24 h, 48 h and 72 h respectively. **a** Large numbers of fluorescence strains were observed on the surface of leaf without orderliness; **b** numerous of bacterial cells were adhered on the epidermal cell groove of tobacco leaves; **c** single cells formed aggregates were colonized the intercellular spaces; **e** numerous fluorescence cells were observed on the surfaces of primary roots; **f** long strings of green fluorescent cells were observed along the root epidermal cells; **g** single GJ-22-gfp cells were observed on root surface and occupied longitudinal intercellular spaces of roots surface. No cells were found in blank control leaf **d** and root **h** of tobacco. Magnification, ×20. And the experiment was repeated seven times

**Fig. 5:**
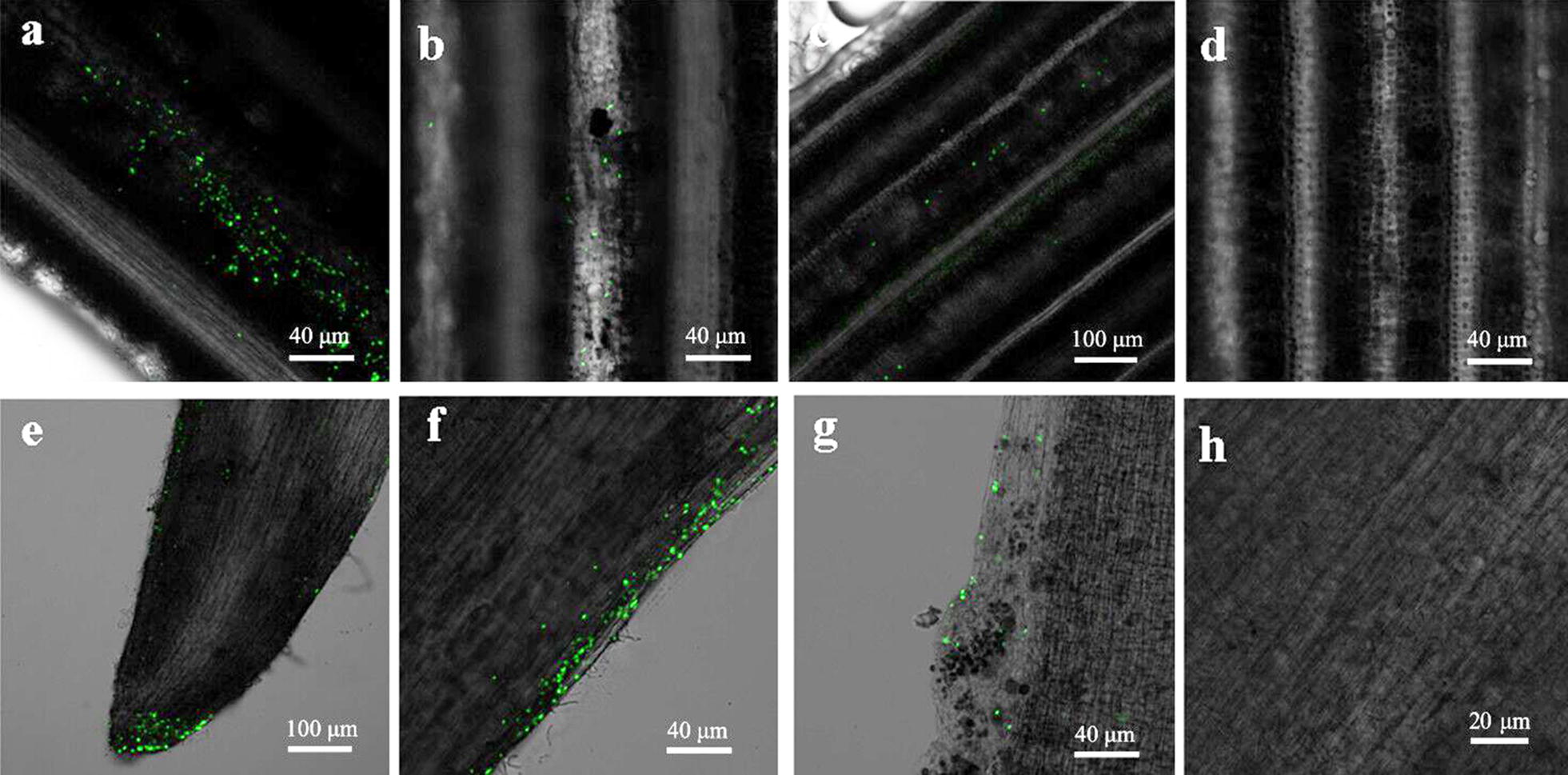
CLSM images of colonization of strain GJ-22-gfp on the leaves and root of rice. **a**–**c** and **e**–**g** were the results of foliar and root treatment for 24 h, 48 h and 72 h respectively. **a** Cells were lined up along the surface layer; **b** cells were observed linear distribution in the leaves damage area throughout the entire length; **c** single cells occupied the longitudinal intercellular space of mesophyll epidermal cells linearly of leaves in rice; **e** cells were seen adsorbed on the root apex, root cap of rice; **f** cells were distributed linearly in the root extension; **g** most fluorescence cells were distributed on the root extension of rice. No fluorescence cells were found on the rice leaf **d** and root **h** with the uninoculated treatment. Magnification: ×20. And the experiment was repeated seven times

The leaves and roots of tobacco and rice were sampled at 24th, 48th and 72nd h after inoculated with GJ-22-gfp. The leaves and root tips of the plants were collected to visualize the expression of strain GJ-22-gfp. The results of CLSM in tobacco and rice were shown in Figs. 4, 5. On the 24th h of inoculation, a large number of bacteria cells were observed to be unevenly accumulated on the surface of tobacco mesophyll cells without any special localization (Fig. 4a). On the 48th h, bacterial cells were adhesion on the epidermal cell groove of tobacco mesophyll cells. However, the complete colonization of the cells were not visualized (Fig. 4b). On the 72nd h sampling detection, numerous single cells formed aggregations and clumps of cells were observed to colonise on the epidermal cells and deep grooves between epidermal cells of leaves of tobacco, and the green fluorescent cells were seen filled with the stomata occasionally (Fig. 4c). And the uninoculated leaves showed no fluorescence cells (Fig. 4d). While on roots, numerous GJ-22 cells were observed on the surfaces of primary roots at the 24th h (Fig. 4e). The distribution of GJ-22-gfp cells on the root surface was random. The cells on roots were less than that of the leaf explicitly. On the 48th h, in addition to individual GJ-22-gfp cells, long strings of green fluorescent cells were also observed along the root epidermal cells (Fig. 4f). Root hairs were seen no GJ-22-gfp cells. While on the final sampling detection, GJ-22-gfp cells were observed on the root surface and occupied between longitudinal intercellular spaces of roots surface (Fig. 4g). No green fluorescent cells were seen in control roots of tobacco (Fig. 4h).

In rice, the CLSM results were shown in Fig. 5. Unlike tobacco, there was no cell aggregation on leaves and roots, high background fluorescence was easily visualized on rice. On the 24th h sampling detection, numerous single GJ-22-gfp cells were lined up along the leaf surface layer of rice (Fig. 5a). However, the results of 48th h sampling showed the bacterial cells on the leaves of rice to be decreased sharply, and the bacterial cells were observed to be in the linear distribution in the leaves damaged area throughout the entire length (Fig. 5b). On the 72nd h, there were only single cells occupied linearly between the longitudinal intercellular spaces of mesophyll epidermal cells (Fig. 5c). And there were no fluorescence cells observed on control treatment (Fig. 5d). On the root of rice, numerous fluorescence cells were seen adhesion on the root apex and root cap of the plant on the 24 h (Fig. 5e). While on the 48 h, most of the cells do not appeared in the apical and coronal of rice root, but found to be distributed linearly in the root extension (Fig. 5f). And the results of the last sampling also showed that most fluorescence cells were distributed on the root extension of rice, and the number of bacteria was reduced significantly (Fig. 5g). Similarly, no fluorescence cells were found on the rice roots of the uninoculated control (Fig. 5h).

### Plant growth promotion and disease resistance test

**Fig. 6:**
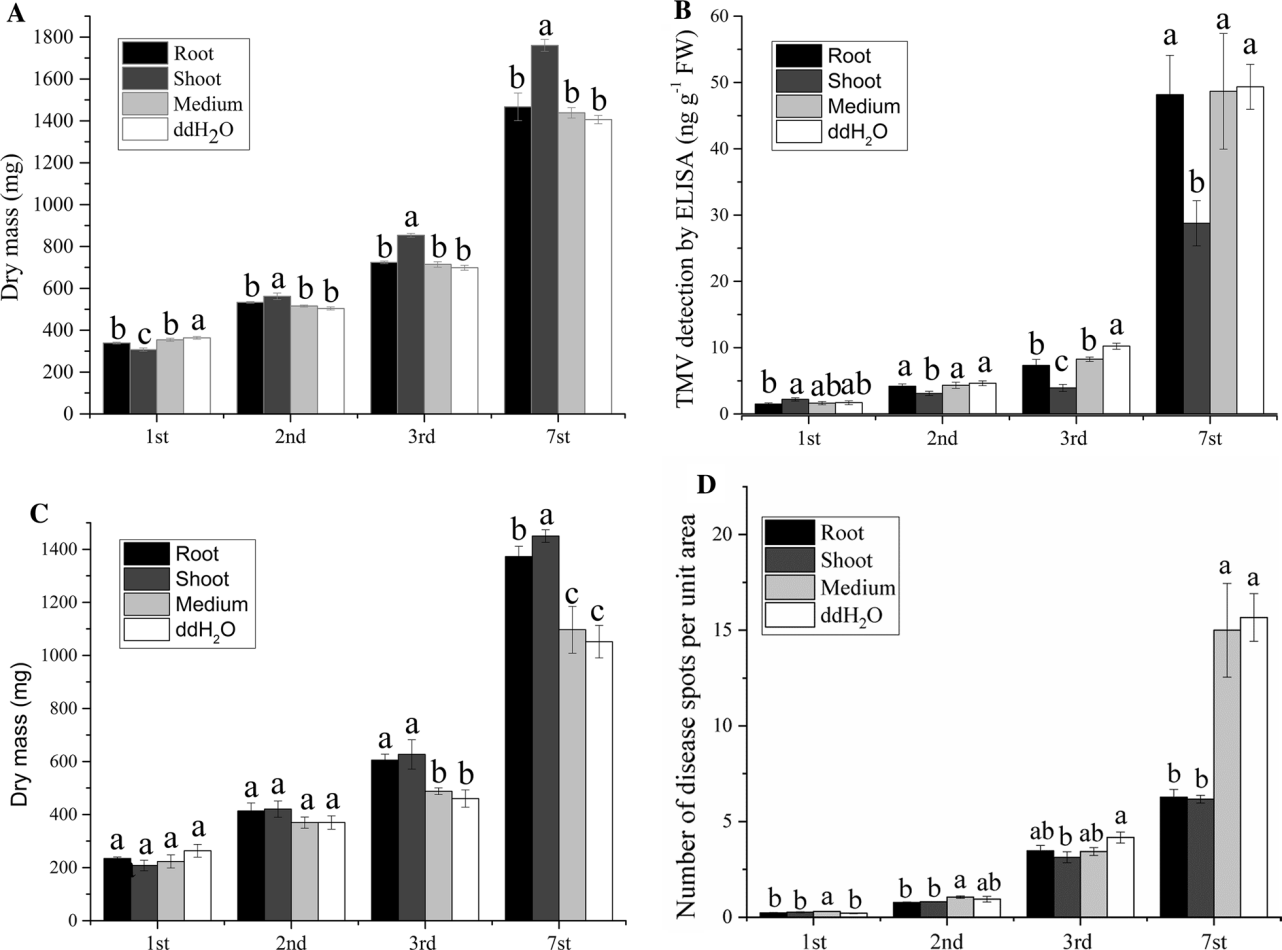
Root and shoot treatment to the growth promotion and Induced resistance test in tobacco and rice. **A** 1 week of tobacco dry mass of four treatments were calculated after 1st, 2nd, 3rd and 7th days after treated. All treatments were repeated 3 times. **B** Determination of TMV accumulation in tobacco by ELISA. The tobacco were pre-treated with the indicated treatment before TMV inoculation. The TMV accumulation was determined by ELISA, and 12 leaf samples were obtained for each treatment. **C** 1 week of rice dry mass of four treatments were calculated after 1st, 2nd, 3rd and 7th days after treated. All treatments were repeated 3 times. **D** Lesion density was performed by counting lesion numbers of unit area. Different letters indicate significantly differences using Fisher’s LSD (P = 0.05)

The dry mass of tobacco (Fig. 6A) and rice (Fig. 6C) treated with the GJ-22-gfp shoot, root, medium and ddH_2_O were weighed. In tobacco, the shoot with GJ-22-gfp suspension were significantly higher than the other treatments from the 2nd days (P < 0.05) (Fig. 6A). In rice, on the 1st and 2nd day of treatment, the treatments with shoot and root were higher than blank control, but no significant (P > 0.05). On the 3rd day, the treatment with shoot and root were higher than medium and ddH_2_O significantly (P < 0.05), and on the sampling of 7th day, the shoot treatment was higher than root and blank control (Fig. 6C).

For the TMV accumulation assay, from the Fig. 6B, with the time over, the TMV accumulation in four treatments increased significantly. On the 1st day of inoculation, the TMV accumulation of shoot treated was higher than other treatments, but on the 2nd day, there had a tendency of the TMV accumulation was lower than root treated and blank control significantly (P < 0.05), and the TMV concentration of root treated was no significantly lower than blank control. While on detection on rice, the GJ-22 treated shoots and roots had no differences with blank control on the 1st and 2nd day. On the 3rd day of inoculation, there has a tendency of reduce the disease plaques, and samplings from 7th day, the lesion number of roots and shoots were less than treated with medium and ddH_2_O significantly (Fig. 6D).

## Discussion

Nowadays, *gfp* is frequently used in biological and biotechnological studies especially as a marker gene (Errampalli et al. 1999; Bloemberg 2007). Koch and co-workers (2001) developed a Tn7-GFP system to express *gfp* in strain *Pseudomonas* for the detection of bacteria cells in the barley rhizosphere. Xiong et al. (2013) produced a GFP-expressing *Rhodococcus* sp. D310-1 strain to study the stability of bacterium in soil and its ability to remove chlorimuron-ethyl from contaminated soils. In 2010, Lagendijk et al. (2010) developed a tool for the transformation of Gram-negative bacteria with a *mCherry* gene and used this fluorescence protein as a marker to visualize bacterial communities in biofilms formed on glass and tomato roots. However, due to the lack of appropriate vectors, no expression vector suitable for expressing foreign genes into *R. palustris*. As a plant growth promoting bacteria, *R. palustris* can not only enhance plant performance under various environment stresses through regulating different plant defense signaling pathways, but also inhibit pathogen infections (Pleban et al. 1995; Shishido et al. 2010; Delany et al. 2001; Nunkaew et al. 2014). To elucidate the mechanism controlling *R. palustris* colonization in plant and to establish a better field application strategy, knowledge and understanding related to *R. palustris* colonization process in its host plant is crucial.

In this study, a broad host range vector pBBR1MCS-2 was used as the basic carrier. A conserved promoter (pckA-P), terminator (pckA-T), and a ribosome binding site (AGGAGG) were added to the basic vector to produce the recombinant vector pBBR1-pckA_PT_-gfp. Before this study, three expression vectors (e.g., PVLT33-GFP, pBBR1MCS-2-pAMP-EGFP and pGreenpuro) were constructed and inserted into *R. palustris* GJ-22 cells, but no fluorescence signal was observed, then the fourth broad host range expression vector pBBR1MCS-2 was constructed based on the earlier reports (Park et al. 2010; Chin A Woeng et al. 2001). Therefore, a suitable promoter is crucial for any expression vector and can decide the efficiency of gene expression.

During the detection of strain stability, 10% of the gfp-labeled strain lost fluorescence after five passages, and the maintenance had fallen to 79% after nine passages. The reason for the disappearance of the fluorescence was still unclear, it was speculated that it is may be a result of the invisible modification and sharing of the foreign gene by the bacteria, which may caused by mismatching or due to lost the *gfp* gene during plasmid replication. In the determination of growth curves of labeled strain and wild-type strain, there was no significant difference in growth, but with the addition of antibiotics, there is a lag phenomenon in the growth stage, which may be the addition of kanamycin that put pressure on the host bacteria metabolism.

*Rhodopseudomonas palustri*s GJ-22, in both cases for the gfp-labeled and the wild type strain, promoted the growth of tobacco and rice, and the effect was related to the hormones secreted by the bacteria (Su et al. 2017; Madhaiyan et al. 2006).

During the colonisation detection, the strain GJ-22 was observed to form aggregates colonised between the mesophyll cells and grooves in tobacco leaves, while in rice, the cells were not formed as the bacterial community as tobacco, but colonised on the longitudinal groove space of epidermal cells. The difference in colonization patterns between tobacco and rice may be attributed to the physiology differences between dicotyledonous and monocotyledonous plant. The differences in colonisation were also reported in methylobacter (Omer et al. 2004; Poonguzhali et al. 2008). It most likely that there were more epidermal cell grooves on the tobacco leaf, as there sites could provide more residual water and variable nutrients to microorganism, similar results were described various of availability compounds, such as, phenol (Sandhu et al. 2007), water (Axtell and Beattie 2002) or the distribution of fructose (Remus-Emsermann et al. 2011). Therefore, there are many different microhabitats offered by leaves. Remus-Emsermann et al. (2014) reported a surprising dense population of 10^4^–10^5^ bacteria mm^−2^ in leaf surface, colonised a wide range of microorganisms, including bacteria, fungi and oomycetes (Agler et al. 2016). And in the other words, the leaf surfaces are the better home to diverse bacterial communities (Remus-Emsermann and Schlechter 2018). The observations by CLSM showed the cells were distributed along the primary roots of tobacco and rice. From the results of CLSM, the population of cells in leaf was far greater than in root, and the number in tobacco was far greater than rice. The dominant colonization location of the bacterial cells could be related to the chemical compounds secreted by bacteria on the leaf surface and the anatomical difference of dicotyledonous plant (Gotz et al. 2006). In addition, the independent treated roots, leaves and stems of tobacco were visualized by CLSM, no fluorescence cells were observed; similarly, the strain GJ-22-gfp was not found in the stems and roots of the leaves independent treated leaves in tobacco. This indicated that the strain GJ-22 could not migrate from the root to the leaf and could not shuttle from the leaf to the root, either. The onset of induced systemic resistance usually achieved in plant growth-promoting bacteria (PGPB), and ISR will be a potential pathway for plant development (Van Loon 2007). The section of ISR induced strains have been greatly promoted worldwide (Wang et al. 2014; Berg 2015), but the difficulties in gfp-tagged photosynthetic bacteria leads to the shortage of biocontrol mechanism. Bacterial aggregates were formed on the mesophyll cells and grooves in tobacco leaves, and showed great growth and disease resistance characteristics, so we hypothesized that strain GJ-22 had the characteristics of inducing plants to produce ISR, which still needs to be verified by a large of experiments. On the other hand, the diversity changes of agricultural ecological environment also brought great changes to the stability of gfp-labeled strain, then the selection of suitable strains to adapt the environment is has a great significance for the development of biocontrol agents.

Generally, the tools of gfp-tagged *R. palustris* have always been a problem for many researchers. In this study, the gfp-labeled strain GJ-22-gfp was constructed successfully and was used for the tracking of *R. palustris* in the plant, which would help to understand the persistence of *R. palustris* in host plants, environment and its contribution to disease resistance and plant growth.

## Supplementary information

**Additional file 1.** Single cell expression of *GFP* tagged strain R. palustris *GJ-22*.

**Additional file 2.** The sequences of gene of gfpmut3a and pckA.

**Additional file 3.** The sequence of recombinant plasmid pBBR1-pckAPT-gfp.

## Data Availability

The strains were available upon request. All data obtained have been included into the manuscript.

## Abbreviations

PBS: phosphate buffered saline
PSB: photosynthetic bacteria
GFP: green fluorescent protein
*R. palustris*: *Rhodopseudomonas palustris*
CLSM: Confocal Laser Scanning Microscopy
pckA: phosphoenolpyruvate carboxykinase
LB: Luria–Bertani
IPTG: isopropyl-d-thiogalactopyranoside
AGE: Agarose Gel Electrophoresis
