# Supplementary material for: A genetic tool for production of GFP-expressing Rhodopseudomonas palustris for visualization of bacterial colonization
Source: AMB Express. 2019 Sep 10;9:141. doi: 10.1186/s13568-019-0866-6 (PMC6737145; doi:10.1186/s13568-019-0866-6)
Supplement: Supplementary file 1 — Additional file 1. Single cell expression of GFP tagged strain R. palustris GJ-22. [file 13568_2019_866_MOESM1_ESM.doc]

**ST 1 Bacterial strains and Plasmids in this S**tudy

| Strains, plasmid | characteristics | Reference |
| --- | --- | --- |
| Strains |  |  |
| *Escherichia coli* DH5α | F^-^ φ80d *lac*ZΔM15 Δ(*lac*ZYA-*arg*F) U169 *end* A1 *rec*A1 *hsd*R17(r_k_^-^ m_k_^+^) *sup*E44λ- *thi*-1 *gyr*A96 *rel*A1 *pho*A | Chen JC et al., 2018 |
| BL21(DE3) Chemically Competent Cell | F- ompT hsdS(rB - mB -) gal dcm(DE3) F | Jeong H et al., 2009 |
| *R. palustris GJ-22* | Gram-negative bacteria | Su P et al., 2017 |
| Plasmids |  |  |
| pBBR1MCS-2 | Wide-host protein expression or cloning vector plasmid | Kovach ME et al., 1994 |
| pBBR1MCS-2-pAMP-EGFP | a broad-host-range cloning vector | Kovach ME et al., 1994 |
| pVLT33 | E. coli/Pseudomonas shuttle vector, oriT, RSF1010, oriV, lacIq, tac promoter, Kmr | De L. V et al., 1994 |
| pGreenpuro | Lentivirus vector | Invitrogen |
| pRK415 | IncP Tc' *lacZa mob* | Keen et al., 1988 |
| pEASY®-T1 | Amp^r^ and Kan^r^, cloning vector, 3,830 bp | TransGen Biotech |

**ST 2 Primers for Amplifying Fragments. The Underlines Were RestrictionS**ites.

| Primers name | Sequence |
| --- | --- |
| pckA-P | F: CGGGGTACCCCGACGGAATGACTTGAGCCAG |
|  | R: GGAAGATCTTCCACCGCGTCGCTGAAGTAAT |
| PckA-T | F: GGAAGATCTTCCCTGTCCACACAATCTGCC |
|  | R: CCGGAATTCCGGCCCTTCGTATTTCGTTCGAT |
| pckA | F: ACGGAATGACTTGAGCCAG |
|  | R: ACCGCGTCGCTGAAGTAAT |
| gfpmuta3 | F: GGAAGATCTTCCATGAGTAAAGGAGAAGAACTTTTC |
|  | R: GGAAGATCTTCCTTATTTGTATAGTTCATCCATGCC |
| PckA+gfpmuta | F: CCGGAATTCCACTATTCAGTCGCCCCA |
|  | R: CCCAAGCTTCTTCGTATTTCGTTCGATCG |
| PckA-P | CGTTCGCCACTATTCAGTCGCCCCACTGGATCAGGGCGGTATATATTGACGTTACCGCGG |
| PckA-T | GCGCGCCGGCGATCGAACGAAATACGAAGGGCGGCCCGCAAGGCCGCCC TT |

**SF 1**

**Single cell expression of *GFP* tagged strain *R.palustris* GJ-22.** (A) Strain GJ-22-gfp. Scale bars: 10μm, Magnification: 60x. (B) the phase picture of A. Scale bars: 10μm, Magnification: 60x. All bacterial cells were grown in broth medium with kanamycin for 4 days under light.

**
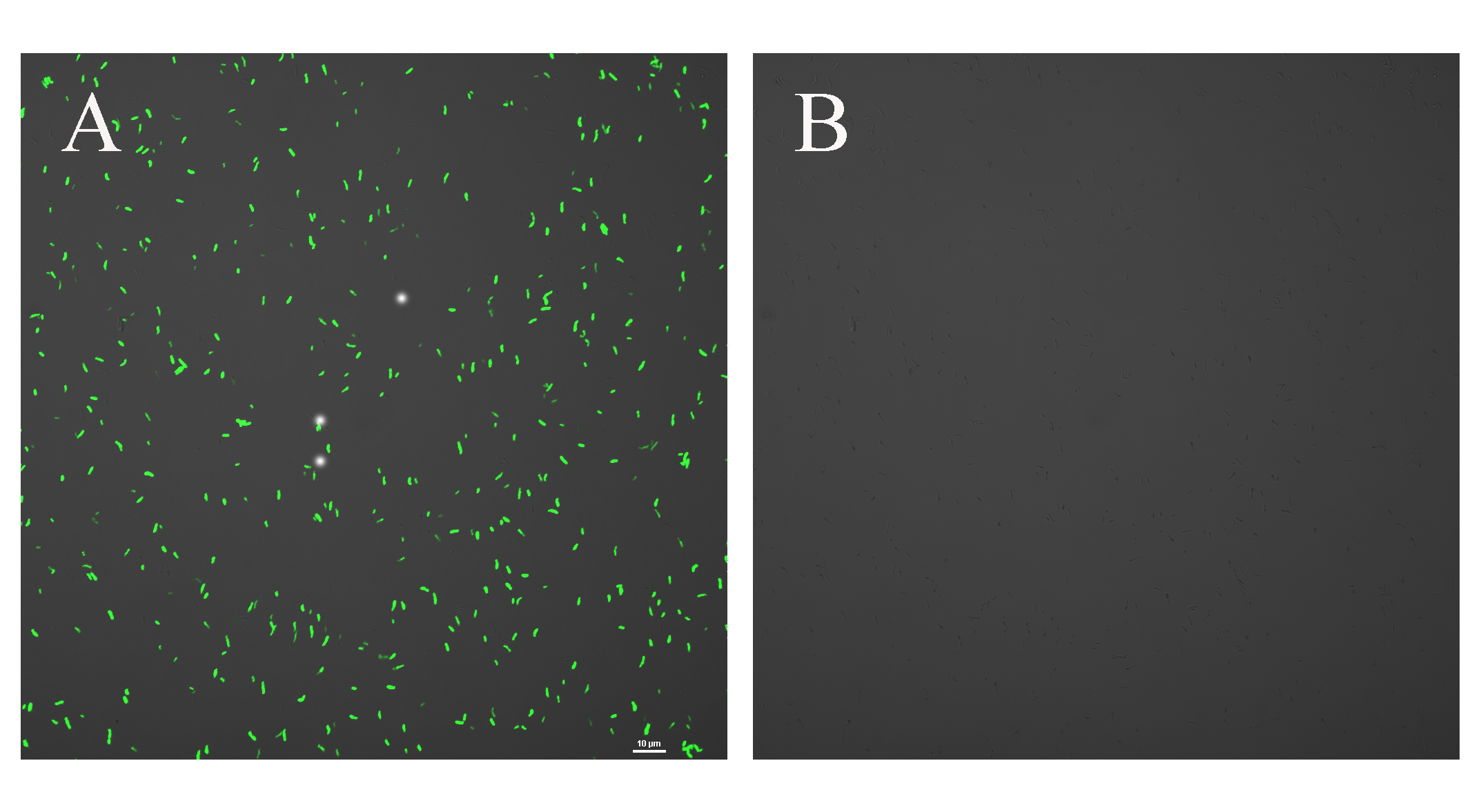
**

**References**

Chen J, Li Y, Zhang K and Wang H (2018) Whole-genome sequence of phage-resistant strain *Escherichia coli* DH5α.Genomea Asm 6(10): e00097-18.

https://doi.org/10.1128/genomeA.00097-18.

De L. V, Eltis L, Kessler, B and Timmis K. N (1993) Analysis of *Pseudomonas*, gene products using *laci q /ptrp-lac,* plasmids and transposons that confer conditional phenotypes. Gene 123(1): 17–24.

https://doi.org/10.1016/0378-1119(93)90533-9.

Jeong H, Barbe V, Lee CH, Vallenet D, Yu DS, Choi SH, Couloux A, Lee SW, Yoon SH, Cattolico L, Hur CG, Park HS, Ségurens B, Kim SC, Oh TW, E. Lenski R, Studier FW, Daegelen P and Kim JF (2009) Genome sequences of *escherichia coli* B strains rel606 and BL21(DE3). J Mole Biol 394(4): 644-652.

https://doi.org/10.1016/j.jmb.2009.09.052.

Su P, Tan XQ, Li CG, Zhang DY, Cheng JE, Zhang SB, Zhou XG, Yan QP, Peng J, Zhang Z, Liu Y and Lu XY (2017) Photosynthetic bacterium *Rhodopseudomonas palustris* GJ-22 induces systemic resistance against viruses. Microb Biotechnol 10(3): 612–624.

https://doi.org/10.1111/1751-7915.12704.

Keen NT, Tamaki S, Kobayashi D and Trollinger D (1988) Improved broad-host-range plasmids for DNA cloning in gram-negative bacteria. Gene 70(1): 191-197.

https://doi.org/10.1016/0378-1119(88)90117-5.

Kovach ME., Phillips RW., Elzer PH., Roop RMII & Peterson KM (1994) Pbbr1mcs: a broad-host-range cloning vector. Biotechniques 16(5): 800.

https://doi.org/10.1002/bip.3603405102.

Su P, Tan XQ, Li CG, Zhang DY, Cheng JE, Zhang SB, Zhou XG, Yan QP, Peng J, Zhang Z, Liu Y and Lu XY (2017) Photosynthetic bacterium *Rhodopseudomonas palustris* GJ-22 induces systemic resistance against viruses. Microb Biotechnol 10(3): 612–624.

https://doi.org/10.1111/1751-7915.12704.
